# Editorial: Host-virus interaction at the omics and ecology levels

**DOI:** 10.3389/fimmu.2023.1209532

**Published:** 2023-05-03

**Authors:** Rúbia Marília de Medeiros, Jacqueline María Valverde-Villegas, Joel Henrique Ellwanger

**Affiliations:** ^1^ Independent Researcher, Porto Alegre, Rio Grande do Sul, Brazil; ^2^ Independent Researcher, Montpellier, France; ^3^ Laboratory of Immunobiology and Immunogenetics, Department of Genetics, Universidade Federal do Rio Grande do Sul (UFRGS), Porto Alegre, Rio Grande do Sul, Brazil

**Keywords:** immunology, immune response, ecology, host-virus interaction, metagenomics, omics, virus, virology

## Introduction

The control of endemic viral diseases is an ongoing challenge in many countries, such as mosquito-borne diseases in Brazil (1) and HIV infection in African nations (2); and emerging and reemerging viruses (e.g., SARS-CoV-2, monkeypox virus) threaten global health (3). The intensification of driving forces of spillover events (transmission of pathogens from animal species to humans) facilitates new viral outbreaks and pandemics. Specifically, environmental changes and social issues (e.g., deforestation, biodiversity loss, bushmeat consumption, unplanned urbanization, intensive farming) increase the chances of humans and animals sharing pathogens (3, 4). Also, the transmission and pathogenesis of viruses are mediated by host components (e.g., immune and genetic factors, microbiome) and viral factors (e.g., transmissibility, replication characteristics, virulence) (5–7), and this information highlights the importance of deciphering host-pathogen interactions in the context of viral diseases.

**Figure 1:**
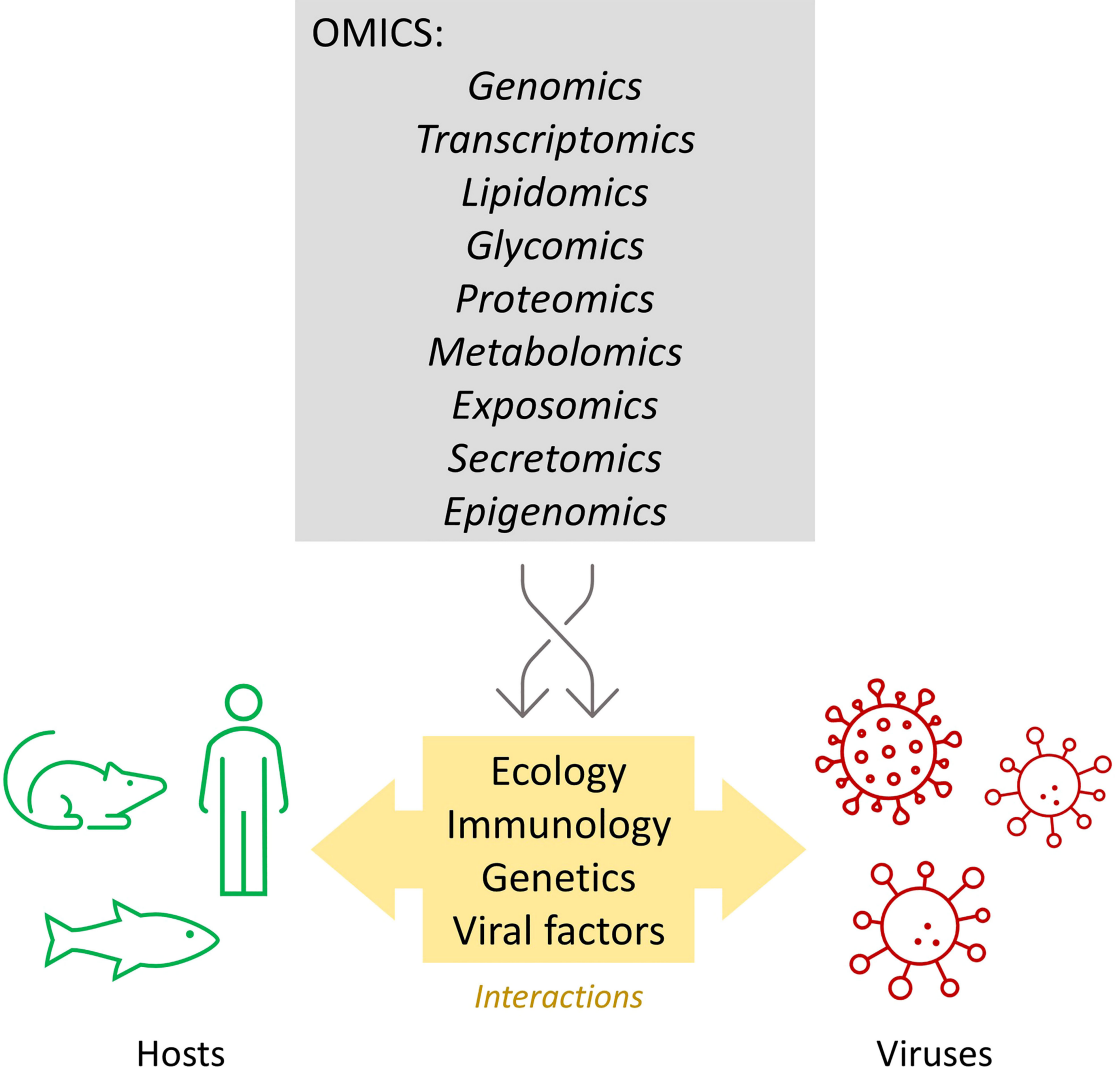
OMICS technologies help decipher ecological, immune, viral, and genetic factors that influence host-virus interactions. Figure created using Microsoft 365.

New challenges demand new approaches. In this context of emerging diseases, OMICS tools (**Figure 1**) can contribute to understanding, tracking, and combating infections more efficiently (8, 9). For example, genomic surveillance of SARS-CoV-2 has played a key role in understanding the transmission dynamics during the COVID-19 pandemic (10). OMICS methods have also contributed to unraveling multiple aspects of host-virus interactions (8, 11, 12). Finally, the dissemination of the One Health concept contributes to the development of infectious disease studies integrating human, animal, and environmental factors (13). Considering recent advances in OMICS tools and viral ecology, this Research Topic highlights studies that have contributed to understanding host-virus interaction at the OMICS and ecology levels.

## New findings

Chikungunya virus (CHIKV) infection is a significant public health issue, especially in developing countries. An adult wild-type C57BL/6J mouse model of CHIKV infection has been used to investigate CHIKV pathogenesis and treatment. However, to what extent the results obtained with this model reflect what occurs in human beings is a matter of debate. Therefore, Bishop et al. performed a robust and elegant comparative transcriptomics analysis using RNA-Seq datasets from the C57BL/6J CHIKV mouse model and datasets from humans (adults and children) infected with CHIKV. In brief, they showed that the C57BL/6J CHIKV mouse model is an important and reliable tool for studying CHIKV pathogenesis and treatment, especially to elucidate the immunological aspects of CHIKV infection.

Different models are used to understand molecular and cellular mechanisms triggered by SARS-COV-2 infection. In this direction, visualizing works on cell lines is important for initial clues on mechanisms and comparing them to what is observed in primary cells. Since the S protein plays a key role in SARS-CoV-2 pathogenesis, a better knowledge of its interaction with host factors is necessary. Thus, Miltner et al. have used THP-1-derived macrophages-like cells to analyze early transcriptomic profile induced by S protein expression. Results suggested several down-regulated genes, such as protocadherins from nervous system development involved in cell adhesion, and type I alpha interferons of the antiviral response. More studies considering other models and functional systems are required to confirm these results.

The N6‐methyladenosine (m^6^A) is the result of enzymatic catalyses of the addition of methyl groups in position 6 of adenine nucleotides, a key component of RNA. This epigenetic modification is essential for RNA processing, transport, and fate, and thus plays a role in gene expression and information flow. Of note, m^6^A is involved in the replication of many viruses and their immune escape process. In this context, Li et al. reviewed the effects of m^6^A on viral replication and how it helps viral RNAs evade recognition by exogenous sensors, such as the gene inducible by retinoic acid 1 (*RIG-1*-like). Moreover, the authors discuss m^6^A as a potential antiviral target.

Pathogenic viruses (e.g., CHIKV, SARS-CoV-2) naturally receive more attention from researchers and the general population due to their impacts on human health. However, most viruses are not pathogenic for humans and even contribute to maintaining ecosystems balance (among many other non-pathogenic biological processes). Many studies in viral genomics are hampered because a significant number of sequences in virome datasets are unknown, composing the so-called “viral dark matter.” Several aspects related to this problem were reviewed by Santiago-Rodriguez and Hollister in a very informative article. The authors pointed out that wet experiments associated with bioinformatic efforts will be necessary to unravel viral dark matter and advance viral metagenomics in different contexts, including human, environmental, and animal health.

Freshwater fish species in aquaculture are exposed to frequent viral outbreaks causing serious diseases impacting aquafarming and ecosystems. This is the case of largemouth bass (*Micropterus salmoides*) when infected by Micropterus salmoides rhabdovirus (MSRV). Fei et al. analyzed the relationship between intestinal microbiota diversity by 16S rRNA gene sequencing and immune-related genes expression by transcriptomic profiling of largemouth bass during MSRV infection. Results suggested a high correlation between the abundance of Streptococcus and the expression of interferon-related genes, when compared control and infection groups, opening new avenues for future functional studies to better understand the microbiota and immune response relationship in these species. In the same direction, Ouyang et al. shed more light on Koi sleepy disease (KSD), an infection caused by carp edema virus (CEV) that threats Koi carp worldwide. Of note, the prevention and control of KSD are hampered by asymptomatic infection cases. Ouyang et al. compared differences in acutely infected and asymptomatic Koi infected based on pathology changes, enzyme and immunoglobulin activities, and host and viral gene expressions. Combining different techniques, the study details genetic, pathology, and immune-related findings concerning CEV pathogenesis and CEV-host interactions. Understanding the host-viral factors underlying diseases in fish species will help to identify biomarkers to diagnose, prevent, and treat fish diseases.

## Conclusion

This Research Topic highlighted the relevance of OMICS tools for the study of host-virus interactions, bringing new information from works that used different samples and experimental approaches. OMICS tools have increasing importance for understanding the complex interactions between pathogens, hosts, and environmental factors.

## Author contributions

RM, JV-V and JE wrote and edited the text. JE prepared the figure. All authors revised and approved the manuscript.
